# Neuroimaging pattern and pathophysiology of cerebellar stroke-like lesions in MELAS with m.3243A>G mutation: a case report

**DOI:** 10.1186/s12883-020-01748-7

**Published:** 2020-05-01

**Authors:** Munenori Oyama, Takahiro Iizuka, Jin Nakahara, Yoshikane Izawa

**Affiliations:** 1 Department of Neurology, Keio University School of Medicine, 35 Shinanomachi, Shinjuku, Tokyo, 160-8582 Japan; 2 Department of Neurology, Kitasato University School of Medicine, Sagamihara, Japan

**Keywords:** MELAS, Stroke-like episodes, Cerebellum, Angiopathy, MRI

## Abstract

**Background:**

Stroke-like episodes (SLEs) in mitochondrial myopathy, encephalopathy, lactic acidosis, and stroke-like episodes (MELAS) with m.3243A > G mutation usually develop in the cerebral cortex. Few reports have documented SLEs in the cerebellum. The clinical neuroimaging features of cerebellar SLEs have not been fully investigated. We report distinctive features of cerebellar stroke-like lesions (SLLs) in a case of MELAS with m.3243A > G mutation.

**Case presentation:**

A 47-year-old Japanese man with type-2 diabetes presented to our hospital with acute onset of aphasia. A brain MRI obtained on admission (day 1) showed increased diffusion-weighted imaging (DWI)/fluid-attenuated inversion recovery (FLAIR) signal in the left anterolateral temporal lobe, which subsequently spread along the cortex posteriorly accompanied by a new lesion in the right anterior temporal lobe. The patient was initially treated with acyclovir and subsequently with immunotherapy. However, on day 45, cerebellar ataxia developed. The brain MRI showed extensive increased DWI/FLAIR signals in the cerebellum along the folia without involvement of deep cerebellar nucleus or cerebellar peduncle; SLLs were incongruent with a vascular territory, similarly to classic cerebral SLLs. Apparent diffusion coefficient (ADC) map did not show reduction in ADC values in the affected folia. Genomic analysis revealed m.3243A > G mutation (heteroplasmy in leukocytes, 17%), confirming the diagnosis of MELAS. After the treatment with taurine (12,000 mg/day), L-arginine (12,000 mg/day), vitamin B1 (100 mg/day), and carnitine (3000 mg/day), the patient became able to follow simple commands, and he was transferred to a rehabilitation center on day 146. The follow-up MRI showed diffuse brain atrophy, including the cerebellum.

**Conclusions:**

SLLs develop in the cerebellum in MELAS with m.3243A > G mutation. The neuroimaging similarities to cerebral SLLs suggest the presence of the common pathophysiological mechanisms underlying both SLEs, which include microangiopathy and increased susceptibility of the cortex to metabolic derangements.

## Background

Stroke-like episodes (SLEs) in mitochondrial myopathy, encephalopathy, lactic acidosis, and stroke-like episodes (MELAS) are episodic events mimicking ischemic stroke [1], and a new definition of SLEs as “epileptic encephalopathy” has recently been proposed [2]. Although the pathogenesis of SLEs remains largely unknown, vascular, metabolic, and neuronal hyperexcitability hypothesis have been proposed [3, 4]. Mitochondrial microangiopathy and neuronal vulnerability to increased energy demand are both presumed to play an important role in the pathogenesis of SLEs [3].

Classic SLEs are usually attributed to a single, continuous lobular edematous lesion that gradually spreads to adjacent cortex beyond the major vascular territory often associated with focal epileptic seizure activity [5, 6]. Stroke-like lesions (SLLs) preferentially involve the cerebral cortex requiring high energy demand and usually spare the basal ganglia. In contrast to classic SLEs, those attributed to sparse or disseminated SLLs confined to the cerebral cortex have recently been described as “non-classic SLEs” [7], suggesting the presence of phenotypical diversity of SLEs associated with m.3243A < G mutation in the mitochondrial tRNA^Leu(UUR)^ gene (*MT-TL1*). Cerebellar SLLs have been regarded as “non-classic SLLs” [7]; however, few studies have documented cerebellar SLLs [5, 7–10]. Accordingly, the clinical neuroimaging features of cerebellar SLEs have not been fully investigated.

We herein report a case of MELAS with m.3243A < G mutation, in which SLLs developed in the cerebellum during the course of classic cerebral SLEs.

## Case presentation

A 47-year-old right-handed Japanese man was admitted to Keio University Hospital with acute onset of sensory aphasia.

Fourteen days before his admission the patient began to have difficulties in operating computers and document processing software. The symptoms worsened over the next 10 days, without headache, fever, or seizure. Three days before his admission, at evening, he suddenly became incoherent and agitated in association with impaired auditory comprehension. He underwent a brain MRI at another hospital; he was suspected of having a herpes simplex encephalitis (HSE), and then he was referred and admitted to the department of neurology at our hospital for further evaluation and treatment. Prior to admission he had no hypoglycemic episodes, preceding viral infection, recent medication changes, or other precipitating events leading to seizure.

He had a past medical history of type-2 diabetes since the age of 41 years, for which he had been treated with acarbose, but he had no other history including cardiomyopathy, atrial fibrillation, migraine, sensorineural hearing loss, seizures, or psychiatric illness. His growth and development were normal. His mother suffered from type-1 diabetes and deafness, which had begun in her 40s, and suddenly died of unknown cause at the age of 68 years. He had no habit of smoking, drinking, or the use of illicit drugs.

On admission (day 1), the temperature was 36.6 °C, the blood pressure 110/65 mmHg, the pulse 84 beat per minute, and the oxygen saturation 97% while he was breathing ambient air. The height was 154 cm, and the weight was 42 kg; the body mass index was 17.7, but physical examination was otherwise unremarkable. On neurologic examination the patient was awake but agitated and uncooperative. He was able to speak fluently but he had paraphasia and preservation; naming, repetition, and auditory and reading comprehension were severely impaired, implying sensory predominant aphasia. Motor and sensory examination was grossly intact. The neck was supple.

**Fig. 1:**
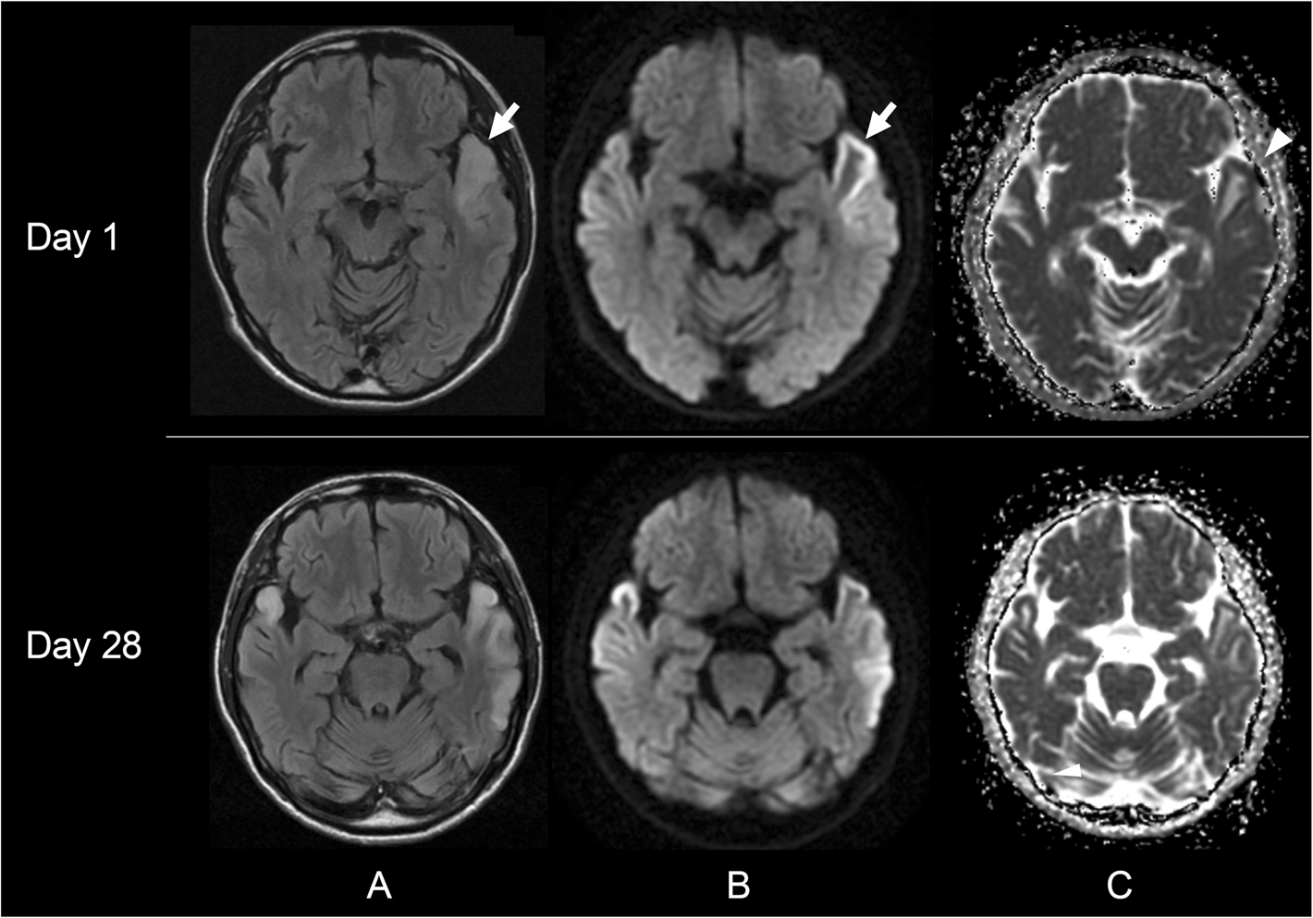
Brain MRIs obtained 14 days after the onset of initial symptom. A brain MRI obtained on admission (day 1) shows increased DWI/FLAIR signal in the left anterolateral temporal lobe, mainly along the cortex with edema (**a**, **b**, arrows). The follow-up brain MRI obtained on day 28 shows a newly appearing increased DWI/FLAIR signal in the right anterior temporal lobe, and posterior spread of the initial left temporal lesion along the cerebral cortex (compatible with classic stroke-like lesion). Note mild reduction in ADC values in the left anterior temporal cortex (**c**, arrowhead), suggesting ongoing regional metabolic derangements. ADC values in the left anterior temporal subcortical white matter is rather increased, suggesting the presence of vasogenic edema. A: DWI; B: FLAIR; C: ADC map

All results of the blood tests on admission were unremarkable, including serum CK level (71 U/L), except an elevated level of HbA1c (7.3%). Cerebrospinal fluid (CSF) examination revealed 1 white blood cell/μL, and a protein level of 46 mg/dl. Oligoclonal bands were negative. A polymerase chain reaction testing was negative for herpes simplex virus. CSF levels of pyruvic and lactic acid were not examined. A brain MRI obtained on admission showed increased diffusion-weighted imaging (DWI)/fluid-attenuated inversion recovery (FLAIR) signal in the left anterolateral temporal lobe mainly affecting the cerebral cortex with underlying subcortical edema (Fig. 1, arrows). Apparent diffusion coefficient (ADC) maps showed mild reduction in ADC values in the left anterior temporal cortex (Fig. 1, arrowheads) suggesting cytotoxic edema, but increase in ADC values in the underlying subcortical white matter suggested vasogenic edema. A MRA did not show vasospasm or vascular occlusion. An electroencephalogram (EEG) recorded on day 4 showed sporadic sharp waves at the left fronto-parietal region.

After admission the patient was empirically treated with intravenous administration of acyclovir and oral levetiracetam (1000 mg/day) for possible HSE. He underwent a whole-body CT and a random skin biopsy but the results were both unremarkable. For possible autoimmune encephalitis, he was also treated with intravenous high-dose methylprednisolone (IVMP: 1000 mg/day, 3 days) on day 10; however, the follow-up brain MRIs obtained on day 7 and day 14 showed a continuous spread of the lesion posteriorly along the cerebral cortex. The follow-up EEG recorded on day 27 showed focal periodic epileptiform discharges (FPEDs) at the left temporal region. The dosage of levetiracetam was increased to 3000 mg/day. However, the brain MRI obtained on day 28 showed a newly appearing lesion in the right anterior temporal lobe (Fig. 1). Because multifocal cortico-subcortical MRI lesions are characteristic features of anti-γ-aminobutyric acid A receptors (GABAaR) encephalitis [11], the patient was also treated with a total of 5 cycles of plasma exchange (PLEX) between day 33 and day 42, without obvious beneficial effects.

**Fig. 2:**
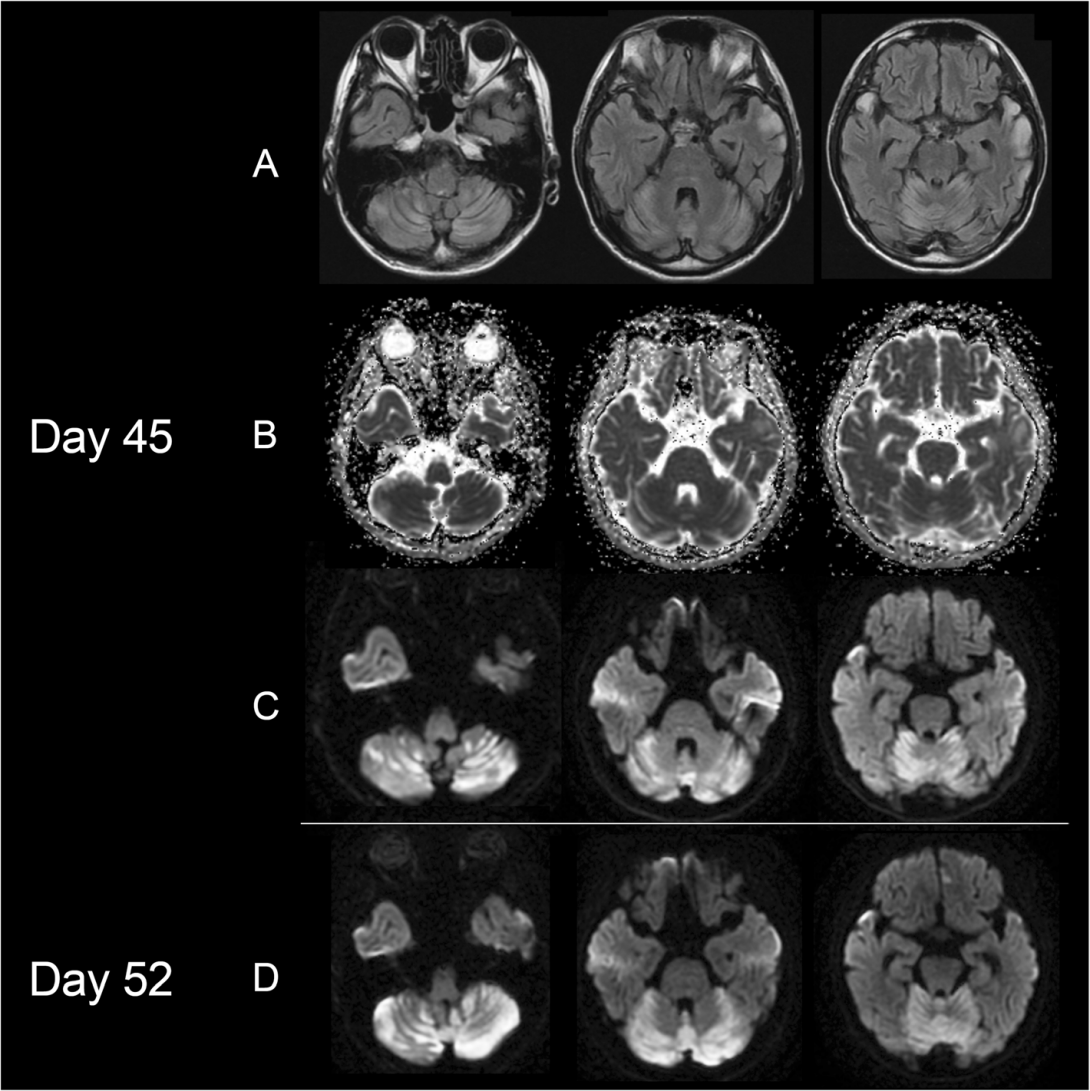
Brain MRIs obtained during the acute stage of cerebellar stroke-like episode. A brain MRI obtained on day 45 (2 days after the onset of dizziness) shows extensive increased DWI/FLAIR signals in the cerebellum, without apparent reduction in ADC values (**a**-**c**). Note preferential involvement of the cerebellar folia sparing deep cerebellar nuclei or cerebellar peduncles, and progressive expansion of edematous lesions along the folia on day 52 (**d**). A: FLAIR: B: ADC map; C and D: DWI

In the evening on day 43 the patient began to complain of feeling dizzy. Two days later dysarthria and cerebellar ataxia became clear. The brain MRI obtained on day 45 showed extensive increased DWI/FLAIR signals in the cerebellum mainly along the folia, without apparent reduction in ADC values (Fig. 2b). A few days later, the patient became mute and somnolent. An EEG revealed FPEDs mainly at the left frontal region. On day 48 CSF lactate and pyruvate levels were 10.2 mmol/L and 0.33 mmol/L, respectively; CSF lactate/pyruvate (L/P) ratios were markedly elevated (30.9). The follow-up brain MRI obtained on day 52 showed expansion of cerebellar lesions, which predominantly affected the folia with edema. Tonsil and nodule were involved, but the deep cerebellar nucleus or cerebellar peduncle were not involved (Fig. 2d).

Based on possible maternal inheritance of diabetes, short stature, elevated CSF L/P ratios, cerebral SLLs, and the lack of evidence of cardiogenic embolism, the patient underwent a genomic analysis, which demonstrated a m.3243A > G mutation with 17% heteroplasmy in leukocytes, confirming the diagnosis of MELAS.

After the treatment with taurine (12,000 mg/day), L-arginine (12,000 mg/day), vitamin B1 (100 mg/day), and carnitine (3000 mg/day), the patient became able to follow simple commands, and he was transferred to a rehabilitation center on day 146. These drugs were well tolerated and there were no adverse events. The brain MRI obtained 4 months after admission showed diffuse brain atrophy including the cerebellum. The modified Rankin Scale at the last follow-up (9 months after admission) was scored 4, with residual cognitive deficits. Nerve conduction study, needle electromyography, and audiometry were not performed during the course of the disease because no consent was obtained for these studies.

## Discussions and conclusions

This case highlights the following important features: (1) autoimmune encephalitis must be diagnosed after exclusion of alternative causes including MELAS; (2) SLLs can develop in the cerebellum in a MELAS patient with m.3243A > G mutation; (3) the cerebellar folia are preferentially involved; (4) distribution of the cerebellar SLLs is incongruent with a vascular territory; (5) deep cerebellar nucleus and cerebellar peduncle are not affected by SLLs.

Despite the absence of pleocytosis, oligoclonal bands, or fever, the patient was initially suspected of having autoimmune encephalitis, HSE, or tumor, based on the initial MRI findings, psychiatric symptoms, and aphasia, resulting in delayed diagnosis. In this case multifocal cortico-subcortical lesions rapidly developed, associated with epileptiform discharges. Similar MRI findings are often seen in patients with anti-GABAaR encephalitis, and SLE-mimicking lesions with focal lactate accumulation and hyperperfusion have recently been reported in a case of anti-GABAaR encephalitis [12]. In MELAS, classic SLLs mainly involve the cortex and gradually spread to the adjacent cortex with accompanying subcortical edema, which is different from anti-GABAaR encephalitis. It should be kept in mind that autoimmune encephalitis must be diagnosed after the reasonable exclusion of alternative causes, including MELAS [13].

It is interesting that the folium (cortex) was preferentially involved in cerebellar SLEs, as was in classic cerebral SLEs. It remains speculative why the cortex is preferentially involved in SLEs of MELAS, but it has been shown that the dendrite-rich cortex are particularly vulnerable to hypoxia [14], thus the cerebral cortex with high metabolic demand are presumed to be more susceptible to metabolic derangements when increase in energy demand (seizure) or decrease in energy supply develop due to ischemia, metabolic breakdown, increased reactive oxygen species, or vasogenic edema associated with blood-brain barrier (BBB) dysfunction, all of which are presumably attributed to respiratory chain dysfunction in neurons, astrocytes, endothelial cells, or smooth muscle cells of small blood vessels [3, 4].

Basal ganglia are often involved in Leigh encephalopathy or MELAS/Leigh overlapping syndrome, but rarely involved by SLLs in MELAS. Interestingly, in this case, the cerebellar cortex, including tonsil and nodule, was extensively involved but deep cerebellar nuclei were not involved on MRI. The reason why such deep structure (basal ganglia or cerebellar nuclei) are being spared in SLLs remains unknown; however, it has been shown that there is a striking increase in number of mitochondria in the smooth muscle and endothelial cells, which were most prominent in the pial arterioles and small arteries but less frequent and severely sparse in the larger pial arteries and intracerebral arterioles and small arteries [15]. The difference in vascular changes in location may in part contribute to difference in neuronal susceptibility to metabolic derangements between the cortex and deep structure.

Although classic SLLs often continuously spread to the adjacent cerebral cortex [5, 6], cerebellar SLLs also showed similar spreading pattern by preferentially involving the folia. Progressive spread of classic SLLs are often accompanied by sustained epileptiform discharges or non-convulsive status epilepticus. In this case, gradual spread of the classic SLLs in the left temporal lobe was accompanied by FPEDs, but it is difficult to assess electrophysiological changes in the cerebellar cortex.

Regarding cerebral perfusion changes, classic cerebral SLLs are often accompanied by focal hyperperfusion reflecting increased synaptic activity [5–7]. A previous one study [9] demonstrated cerebellar hyperperfusion during the acute stage of cerebellar SLEs, but unfortunately, we did not assess perfusion changes.

Cerebellar ataxia is one of the common symptoms in m.3243A > G carriers (20–66%) [16, 17] and cerebellar atrophy is also seen in 24% of patients with MELAS [16]. However, in most cases ataxia is associated with chronic neurodegenerative process of the cerebellum (atrophy), not due to acute onset of cerebellar SLEs. Accordingly, cerebellar SLEs have rarely been reported. Although in this case we cannot exclude the possibility that cerebellar SLEs were precipitated by a preceding PLEX, the pathogenesis of cerebellar SLEs remain to be fully elucidated. The lack of apparent reduction in ADC values may suggest that the cerebellar SLLs mainly consist of vasogenic edema as reported in classic cerebral SLEs [18]. BBB dysfunction has also been implicated in the mechanism of cerebellar SLLs based on extravasation of plasma protein in the small blood vessels in the cerebellum in patients with MELAS [9].

This study has limitations of being retrospective and based on a single case report. We did not perform a perfusion study or histopathological examination of the affected cerebellum.

Despite these limitations, we could characterize several distinctive features of cerebellar SLEs in a patient with m.3243A < G mutation. Clinical neuroradiological similarities of cerebellar and cerebral SLEs may imply the presence of the common pathophysiological mechanisms underlying both SLEs. We stress on a potential role of microangiopathy and increased susceptibility of the cerebellar cortex to metabolic derangements, as previously reported in classic cerebral SLEs [5, 6]. Further studies are required to conclude the pathogenesis of cerebellar SLEs.

## Data Availability

Data sharing is not applicable to this article as no datasets were generated or analyzed during the current study.

## Abbreviations

ADC: Apparent diffusion coefficient
BBB: Blood brain barrier
CSF: Cerebrospinal fluid
DWI: Diffusion-weighted imaging
EEG: Electroencephalogram
FLAIR: Fluid-attenuated inversion recovery
FPEDs: Focal periodic epileptiform discharges
GABA_A_R: γ-aminobutyric acid A receptors
IVMP: Intravenous high-dose methylprednisolone
MELAS: Mitochondrial myopathy, encephalopathy, lactic acidosis, and stroke-like episodes
MRA: Magnetic resonance angiography
MRI: Magnetic resonance imaging
PLEX: Plasma exchange
SLEs: Stroke-like episodes
SLLs: Stroke-like lesions
